# Reviewers in 2021

**DOI:** 10.1098/rsob.220055

**Published:** 2022-03-16

**Authors:** Jon Pines

The Editors of *Open Biology* would like to thank all reviewers who contributed their time by refereeing manuscripts submitted throughout 2021. From previous feedback, we know that most reviewers value recognition of their efforts by the journal and through services, such as Publons, which is integrated with *Open Biology*.

To provide an opportunity for recognition, we list the names of all reviewers who opted in. This article is made permanent and citable by its digital object identifier (DOI). We encourage you to quote your contribution in your applications for tenure and grants or other forms of research assessment. Where you have provided it, we have included your ORCID, so your contribution can be unambiguously assigned to you.

The team really does appreciate all the work our reviewers do on behalf of the journal, and we look forward to working with you again in future.

Acosta-Serrano A https://orcid.org/0000-0002-2576-7959
Ahmad K https://orcid.org/0000-0002-1095-8445
Aldred N https://orcid.org/0000-0003-2285-5133
Ali F
Allen A
Alvarsson A https://orcid.org/0000-0003-0868-8578
Andersen J https://orcid.org/0000-0002-4395-5232
Arimura Y
Azem A
Ballestrem C
Barrachina F
Berridge M
Besteiro S https://orcid.org/0000-0003-1853-1494
Beynon R https://orcid.org/0000-0003-0857-495X
Bharti P
Bin Z
Bin Z
Bizard A
Blanco-Aparicio C
Bloom K
Bodas M
Boschetti C
Bose R
Boukhatmi H
Brown N
Buchwalter A
Bulgakova N https://orcid.org/0000-0002-3780-8164
Bunch H https://orcid.org/0000-0003-0038-1985
Caceres J
Callaini G
Camins A
Campbell C
Cancio I
Čapková Frydrychová R https://orcid.org/0000-0003-2372-4848
Carmena A
Casali A
Catic A https://orcid.org/0000-0002-3572-7598
Chamberlain L https://orcid.org/0000-0002-8701-4995
Chan K-Y https://orcid.org/0000-0002-5974-1650
Chen F
Chen X
Chen X
Cheng C-W
Cheng Y-C
Claereboudt E
Claessen D https://orcid.org/0000-0002-0789-2633
Cohen N
Colgren J
Contreras O https://orcid.org/0000-0002-8722-9371
Cooper C https://orcid.org/0000-0002-9197-8041
Crow A
Cyert M
D'Arcy P https://orcid.org/0000-0001-6671-7600
Deschenes R
Dougan S https://orcid.org/0000-0002-2263-363X
Du L-L https://orcid.org/0000-0002-1028-7397
Ducatelle R
Duclos M https://orcid.org/0000-0002-9305-3982
Dundee M
Dyson P
El-Danaf R
Elia M
Engstler M https://orcid.org/0000-0003-1436-5759
Erhardt S
Estévez R
Etich J https://orcid.org/0000-0003-3238-6692
Farge G
Field C
Field M
Fiorani F
Fisher D
Fisher R
Forth J-H
Franco S
Fukagawa T
Fukata M
Fuller W https://orcid.org/0000-0002-5883-4433
Funabiki H https://orcid.org/0000-0003-4831-4087
Galasko D
Gallego-Delgado J https://orcid.org/0000-0002-8914-7740
Ganz J
Gardlik R
Gazouli M
Gershenzon J
Godinho S
Gondim K
Gravholt C
Gray R https://orcid.org/0000-0001-9668-6497
Griffiths G
Grose R https://orcid.org/0000-0002-4738-0173
Gruneberg U
Hadjantonakis A-K https://orcid.org/0000-0002-7580-5124
Hammarlund-Udenaes M
Hamza I
Harwood J
Hemler M
Hiraoka Y
Hochegger H
Holehouse A
Holmes M
Holthoff K
Hoskisson P https://orcid.org/0000-0003-4332-1640
Hougland J https://orcid.org/0000-0003-0444-1017
Hunter T
Ilan N
Inestrosa N
Isokawa M
Itzhaki LS
Jadavji N
Jakimowiz D https://orcid.org/0000-0002-7857-064X
Jin W
Jin Z
Julien E
Kalsbeek A
Kathayat R
Kawaguchi S-Y
Kawashima S
Ke H
Keller S
Kerr I D
Khan FH
Kim C
Kim JY
Kim JH https://orcid.org/0000-0002-6776-2451
Kloxin AM
Koletas E
Kuan Y-S
Kuil L
Kurita T https://orcid.org/0000-0003-4281-5884
Lackner K
Lanyon-Hogg T
Lax G
Leigh N
Liang S https://orcid.org/0000-0001-7317-5651
Lin G https://orcid.org/0000-0001-8097-7222
Loppin B https://orcid.org/0000-0002-7166-9233
Lu Z https://orcid.org/0000-0002-7803-8442
Luty AJF
Madrid M
Makumi A
Marasco D
Marco A
Martin F
Martinez P
Martinho R
Massana R
McDougall A
Knafo S https://orcid.org/0000-0002-4478-7584
Min R
Moran Y https://orcid.org/0000-0001-9928-9294
Morrow Z
Mullin JM
Netherton C
Nichols J
Nielsen O
Nodwell J
Norton L
Odone A
Pawelec G
Pestov D https://orcid.org/0000-0002-1425-9879
Pfanner N
Plotkin M
Polishchuk R
Polymenis M https://orcid.org/0000-0003-1507-0936
Ranjit M
Ravindran V
Rhind N
Rigden D
Rikhy R
Robinson D
Roig I
Rudolph C https://orcid.org/0000-0003-2493-3748
Sadej R https://orcid.org/0000-0003-2583-7299
Sakai D
Sapio R
Sanes J
Sango K
Santocanale C
Sarkar DS https://orcid.org/0000-0002-0311-0710
Sarko D
Sauer J-D
Scheele C https://orcid.org/0000-0001-8999-5451
Schniepp HC
Semenkovich C
Seutin V
Shcherbata H
Sheng N
Shenolikar S
Shu X
Shutt T
Sidhu G
Sobrado P
Soldati-Favre D
Spencer S
Stülke J
Sudhof T
Susi P
Swan A
Swelum AA-A
Tan K
Tanaka K
Tandon R
Tate E
Thatcher GRJ https://orcid.org/0000-0002-7757-1739
Thomas G
Tirloni L
Todd R
Tommasino M http://orcid.org/0000-0001-5779-2449
Tsubouchi H
Utrilla J https://orcid.org/0000-0003-3048-9241
van der Giezen M https://orcid.org/0000-0002-1033-1335
van der Goot G
Vega JL
Veit M
Vendetti S
Vincent B
Vlodavsky I
Wang F https://orcid.org/0000-0001-8730-0003
Wang Q
Weissman K
Weller S
Westermann S
Wilkie I
Williams RS https://orcid.org/0000-0002-4610-8397
Witte OW
Wolfner M
Yan A
Yan B
Yuan X https://orcid.org/0000-0002-0969-5748
Zanetti G
Zechiedrich L
Zhang B
Zhang F
Zhang X https://orcid.org/0000-0001-9733-8494
Zhao Y
Zhu G
Zille M https://orcid.org/0000-0002-0609-8956

